# Potential implications of long-acting GLP-1 receptor agonists for critically ill

**DOI:** 10.1186/s13054-024-04945-9

**Published:** 2024-05-13

**Authors:** Luping Wang, Hao Yang, Xiaoxiao Xia, Bo Wang, Qin Wu

**Affiliations:** Department of Critical Care Medicine, West China Hospital, Sichuan University, Chengdu, China

Dear editor,

The increasing prevalence of obesity and type 2 diabetes has led to a surge in the use of long-acting glucagon-like peptide-1 receptor agonists (GLP-1 RAs). While these medications are approved for the treatment of type 2 diabetes and obesity, their use has important implications for intensivists, as critically ill patients admitted to the intensive care unit (ICU) may have been receiving these medications prior to admission.

Given the prolonged half-lives of long-acting GLP-1 RAs, such as semaglutide (half-life of approximately 7 days) [1], their effects may persist throughout a patient's ICU stay, even if the medication is discontinued upon admission. This prolonged action can have significant implications for the management of these patients, particularly in terms of gastric emptying, enteral nutrition tolerance, and glycemic control.

Recent literature has highlighted the potential impact of GLP-1 RAs on gastric emptying in patients under anesthesia. Sen and colleagues reported that patients who were taking GLP1 RAs prior to surgery had larger cross sectional areas of the gastric antrum than those who weren’t taking GLP1 RAs [2]. Whether this has any impact on the risk of aspiration is unknown [3]. It is important that intensivists understand that short-acting GLP-1 RAs have a greater impact on gastric emptying than long acting GLP 1 RAs [4]. While GLP-1 has a marked glucose lowering effect in the critically ill [5], which is mediated, at least in part by slowing gastric emptying, this occurs when gastric emptying is relatively normal, but not when it is delayed [6]. Prokinetic drugs, such as erythromycin, counteract the slowing of gastric emptying that occurs with GLP-1 [7].

Furthermore, the impact of long-acting GLP-1 RAs on glycemic control in critically ill patients remains poorly understood. GLP-1 RAs attenuate stress-induced hyperglycemia [8], with a reduced risk of hypoglycemia when compared to insulin administration [9], but the effects in patients with renal failure are unknown [10].

As the use of long-acting GLP-1 RAs continues to expand, intensivists must be prepared to manage the unique challenges these medications may pose in the critical care setting. We call for further research to better understand the impact of GLP-1 RAs on critical care outcomes. Additionally, we urge the development of evidence-based guidelines to assist clinicians in managing patients receiving these medications in the ICU, with a focus on monitoring strategies, dose adjustments, and the potential need for alternative management approaches.

In conclusion, the increasing use of long-acting GLP-1 RAs prior to ICU presents unique challenges for intensivists managing critically ill patients. By addressing the current knowledge gaps and developing evidence-based guidelines, we can optimize the care of these patients and improve their outcomes in the ICU.

## Data Availability

Not applicable.

## Abbreviations

GLP-1 RAs: Glucagon-like peptide-1 receptor agonists
ICU: Intensive care unit
